# Drosophila melanogaster as a model organism for Alzheimer’s disease

**DOI:** 10.1186/1750-1326-8-35

**Published:** 2013-11-22

**Authors:** Katja Prüßing, Aaron Voigt, Jörg B Schulz

**Affiliations:** 1 Department of Neurology, University Medical Center, RWTH Aachen, Pauwelsstrasse 30, D-52074 Aachen, Germany; 2 Jülich-Aachen Research Alliance (JARA) Brain, 52074, Aachen, Germany; 3 EURON - European Graduate School of Neuroscience, Aachen, Germany

**Keywords:** *Drosophila melanogaster*, Amyloid-β, Tau, Alzheimer’s disease

## Abstract

*Drosophila melanogaster* provides an important resource for *in vivo* modifier screens of neurodegenerative diseases. To study the underlying pathogenesis of Alzheimer’s disease, fly models that address Tau or amyloid toxicity have been developed. Overexpression of human wild-type or mutant Tau causes age-dependent neurodegeneration, axonal transport defects and early death. Large-scale screens utilizing a neurodegenerative phenotype induced by eye-specific overexpression of human Tau have identified several kinases and phosphatases, apoptotic regulators and cytoskeleton proteins as determinants of Tau toxicity *in vivo*. The APP ortholog of *Drosophila* (dAPPl) shares the characteristic domains with vertebrate APP family members, but does not contain the human Aβ42 domain. To circumvent this drawback, researches have developed strategies by either direct secretion of human Aβ42 or triple transgenic flies expressing human APP, β-secretase and *Drosophila* γ-secretase presenilin (dPsn). Here, we provide a brief overview of how fly models of AD have contributed to our knowledge of the pathomechanisms of disease.

## Background

Alzheimer’s disease (AD) is the most common irreversible cause of dementia. It is characterized by cognitive impairment and progressive neurodegeneration and affects more than 24 million people worldwide [1]. With AD diagnoses being on the rise, burdening existing healthcare support mechanisms, the disease is set to wreak havoc on the healthcare industry. Definite diagnosis of AD requires the correct identification of classical neuropathological hallmarks, which are extracellular amyloid plaques and intracellular neurofibrillary tangles.

Plaques are primarily composed of Amyloid-β peptides (Aβ) generated by differential proteolytic cleavage of the transmembrane receptor Amyloid Precursor Protein (APP). The endoproteolysis is performed by the β-site APP-cleaving enzyme (BACE) and γ-secretases, consisting of Presenilin 1/2, Nicastrin, APH-1 and PEN-2 [2]. Among other peptides and proteins, the two cleavage products Aβ40 and Aβ42 are found in plaques. However, Aβ42 is the predominant form and is considered to be the main amyloidogenic peptide as it forms fibrils more easily [3].

The neurofibrillary tangles are composed of hyperphosphorylated Tau proteins and are found intracellularly in affected neurons. In non-disease situation, Tau is bound to microtubuli (MT) and thereby leads to the stabilization of MT. The affinity of Tau to MT is regulated by phosphorylation of Tau’s MT binding sites. A high degree of phosphorylation results in detachment from MT and subsequent Tau aggregation, finally causing the formation of neurofibrillary tangles [4].

The dominating, but not exclusive explanation for the molecular basis of AD pathology is the amyloid cascade hypothesis. It states that the deposition of Aβ in the brain is the central event initiating disease progression [5]. Aβ deposits activate downstream neurotoxic mechanisms including deregulation of Tau-MT-binding properties.

The amyloid cascade hypothesis is supported by the fact that mutations implicated in familial AD are known to increase ratios of Aβ42/Aβ40 and aggregation [6-8]. Although Tau mutations lead to neurodegeneration [9], none of the disease-linked Tau mutations is linked to familial AD. Mutations in Tau rather cause fronto-temporal dementia or progressive nuclear palsy in which Aβ42 deposits are absent [10].

Several lines of evidence support the idea that Tau acts downstream of Aβ42 toxicity. Clearance of Aβ reduced early hyperphosphorylated Tau aggregation in double transgenic mice, whereas increasing Tau burden did not affect Aβ42 accumulation [11]. Furthermore, it is known that reduction of Tau protein levels leads to an amelioration of Aβ-induced learning and memory impairment [12]. Mechanisms linking extracellular Aβ42 to intracellular Tau are a subject of intensive research. One possible molecular mechanism is associated with a dendritic function of Tau [13]. Dendritic Tau targets Fyn kinase to postsynaptic density, where Fyn facilitates stabilization of a complex triggering downstream excitotoxic signaling [13].

In modern research several model systems have been developed trying to reveal molecular mechanisms linking pathological hallmarks like aggregating Tau and Aβ peptides to neurodegeneration finally resulting in progressive memory loss as observed in AD. However, key features of the disease etiology still remain elusive and no efficient therapy has been found so far.

This review summarizes the utilization of *Drosophila melanogaster* to mimic AD pathology inflicted by excess Tau protein and Aβ42 peptide production.

### Drosophila as a model organism for AD

Animal model systems are used to study specific functional aspects of human diseases in general and neurodegenerative diseases in particular. AD models range from yeast [14] and *Caenorhabditis elegans*[15] to mammals and human cell culture systems [16-18]. However, no model system combines easy use and essential criteria of AD, like cognitive and behavioral dysfunction caused by cell type-specific neurodegeneration, cellular pathophysiology including aggregate formation, clear pattern of inheritance and genetic homogeneity. Although vertebrate model organisms reflect pathologic hallmarks of human diseases very well, these model organisms have the disadvantage of care, time and cost-intensive handling. Using comparable short-lived model organisms allows fast data acquisition facilitating large-scale experiments, although these organisms might lack some pathophysiological characteristics of AD (a summary of invertebrate AD models is provided in [19]).

*Drosophila* has more than a hundred-year history in genetic research [20]. It is used as prime model organism for experimental studies of multi-cellular eukaryotic biology and it combines genetic, anatomic, behavioral, methodical and even economic advantages. It is one of the first organisms with a fully sequenced genome [21]. Approximately 13,600 protein-coding genes are located in only four chromosomes. The fly anatomy is well studied, its brain and nervous system are quite complex [22]. Its anatomical features like the compound eye allow easy access for phenotypic characterization. The fly’s behavior ranges from simple avoidance to learning and memory [23]. Due to its long history as an animal model in research, a wide variety of well-established molecular genetics tools are available [24]. Another advantage regarding its usefulness in biomedical research, especially in the field of neurodegenerative diseases, is its short lifespan. Depending on diet and stress it ranges up to an average maximum of 120 days. All this makes *Drosophila* an ideal organism to study neurodegenerative diseases like AD [25]. Previous studies have clearly shown that the expression of disease-related gene products (Tau protein and Aβ42 peptide, respectively) causes phenotypes in flies. Reminiscent of the situation observed in AD patients, flies show a robust decline of neurons upon Aβ42 and/or Tau overexpression. Depending on the neuronal subset the expression of the AD-linked peptides/proteins is targeted to, the neuronal decline has different phenotypic outcomes like early death, reduced locomotion in larvae and adults, decreased flight ability, blindness, rough eye texture, etc. All these parameters can be analyzed and quantified, thus making the fly a reasonable organism to study specific aspects of AD pathology. In addition, more sophisticated behavioral or cognitive assays can be performed in flies. Applying such assays on fly models of AD, a decline in cognition, a hallmark of AD was observed. Overall, the fly is a powerful model to study the molecular basis of neuronal decline in the context of AD [26,27]. Tests on alterations in behavior and/or cognition are possible in flies. However, their analysis is often time-consuming and the conclusions that can be drawn with regard to humans are fairly limited. An overview of advantages and disadvantages using *Drosophila* as a model organism to study neurodegenerative diseases like AD is provided in Table 1.

**Table 1:** **Advantages and disadvantages of using *Drosophila *** **as a model organism for neurodegenerative diseases like AD**

**Advantage**	**Disadvantage**
No ethical problems/no restrictions according to animal protection laws	Brain anatomy, cardiovascular system and respiration systems differs substantially from humans
Easy and cheap to maintain in large quantities, time and cost effective handling	No easy measure of complex behavior
Genetic manipulation is fast and inexpensive (3 month, < $ 500 per transgene)	Only basic measures of cognitive decline
Plethora of available resources/stocks (e.g. genome-wide RNAi-library)	Sometimes poor conservation of proteins/protein function
Short generation time (~10 days), short life span (2–3 month) - > easy to use for screens	Maintenance as living cultures only, no permanent conservation (e.g. frozen stocks) possible
Fully sequenced and annotated genome	Less complex and adaptive immune system as in vertebrates
Good conservation of basic signaling pathways and cellular processes in general	Effects of drugs on the organism might differ strongly (e.g. conversion of pro-toxins to toxins in liver)
Low redundancy/reduced number of paralogous genes compared to vertebrates	
Probably best analyzed/understood multi- cellular organism	
More complex organism compare to *C. elegans* and yeast	
Balancer chromosomes allow the maintenance of mutations/trangenes without genotyping	

### Drosophila models for Aβ toxicity

Comparative analysis of whole genomes revealed striking similarities between structural composition of human and *Drosophila* genes [28]. Nearly 70% of human disease-causing genes have orthologs in the fly [29]. Given this, it is not surprising that orthologs associated to known AD genes not only exist in *Drosophila*, but also exhibit functional conservation.

*Drosophila* harbors an APP ortholog [30] and all components of the γ-secretase complex [31]. Although a β-secretase-like enzyme was identified in flies [32], it displays very low β-secretase activity [33]. The *Drosophila* APP ortholog dAPPl shares the characteristic domains with vertebrate APP family members [30]. However, the region corresponding to the Aβ peptides lacks significant homology [30]. As a consequence, there is no endogenous Aβ production in the fly. Nevertheless, overexpression of the β-secretase-like protein resulted in cleavage of dAPPl producing a fragment corresponding to the human Aβ peptide [32]. Interestingly, this fragment is also able to aggregate and induces age-dependent behavioral deficits and neurodegeneration [32].

In addition to endogenous Aβ production, transgenic flies have been generated to study human Aβ42-induced toxicity and neurodegeneration [34-37]. Greeve and co-workers generated a triple transgenic fly expressing human APP (hAPP), human β-secretase (hBACE) and *Drosophila* γ-secretase presenilin (dPsn) with point mutations corresponding to familial AD mutations N141I, L235P and E280A [36,38]. These flies developed age-dependent neurodegenerative phenotypes such as photoreceptor cell loss, severe degeneration of their projecting axons and early lethality. Co-expression of hAPP and hBACE favored the processing of a higher glycosylated species of hAPP in *Drosophila* resulting in Aβ40 and Aβ42 peptide forming plaques in transgene expressing tissue. Plaque deposition precedes the onset of neurodegeneration and coexpression of mutant dPsn results in acceleration of photoreceptor degeneration [36]. The described triple transgenic model clearly demonstrates the similarities between the biochemical pathways induced by Aβ42 deposition in flies and humans.

**Figure 1:**
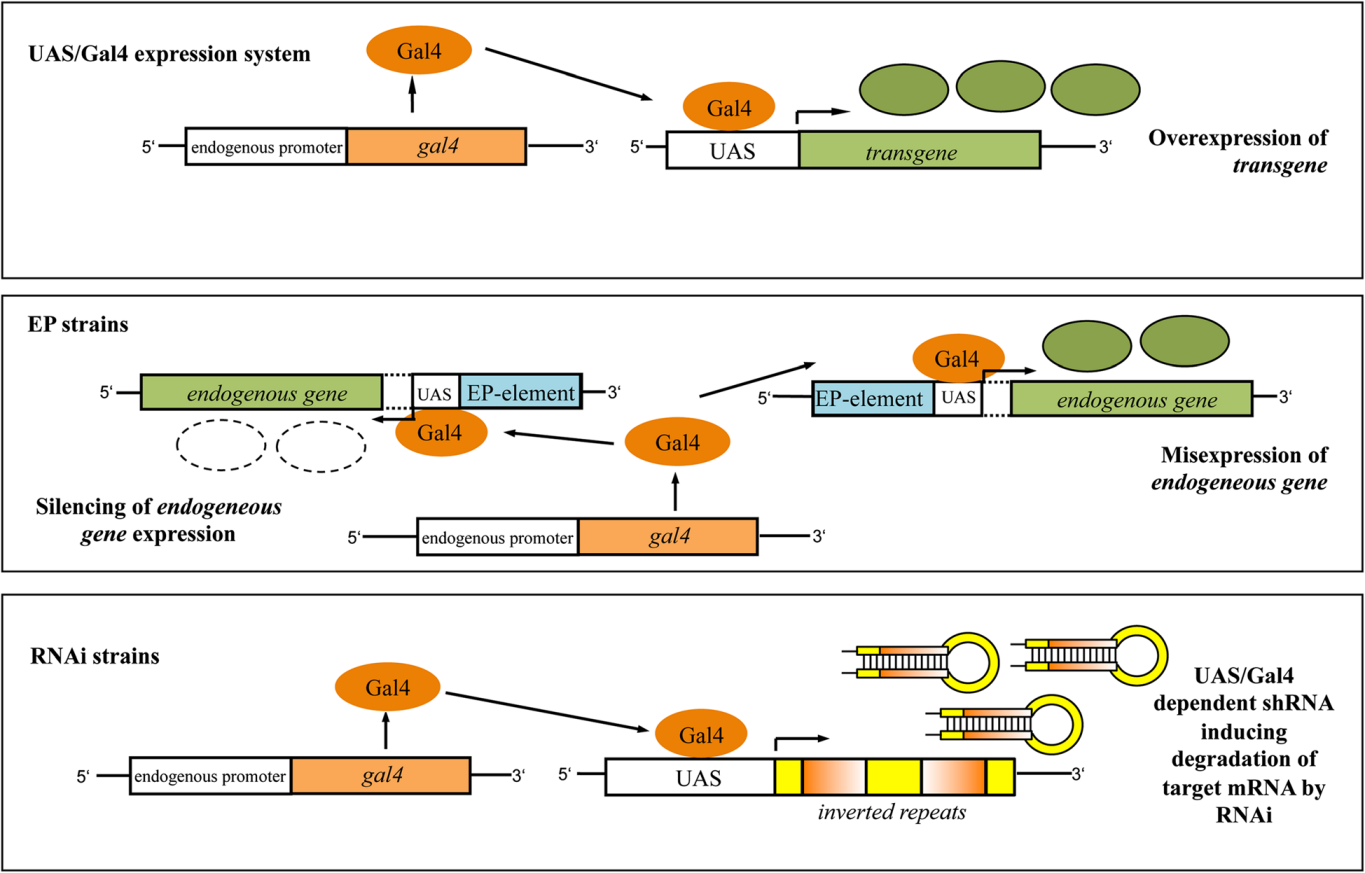
**Genetic tools in *Drosophila.*** In *Drosophila* the **UAS/Gal4 expression system** has been used extensively to express endogenous and exogenous sequences in the tissue of interest [39]. This is implemented using two different lines. The so-called driver line contains a Gal4 coding sequence inserted downstream of a promoter of an endogenous *Drosophila* gene. Gal4 is a transcription factor originating from *Saccharomyces cerevisiae*[40]. It specifically binds to promoter elements termed upstream activating sequence (UAS), thus activating expression of the downstream target sequence [40,41]. A collection of Gal4 driver lines which display a great variety of Gal4 expression in numerous tissues and organs is available to the public [42]. Frequently used are the *glass multimer reporter* (GMR) driver inducing retinal expression [43] and the *elav* driver inducing pan-neuronal expression [44]. After crossbreeding both, the Gal4 driver and the UAS line, the UAS target sequences will be expressed in a spatiotemporal manner (depending on the Gal4 driver used). **EP-elements** are randomly inserted in the fly genome and contain UAS sites. Depending on the orientation EP-elements might facilitate activation (same orientation) or inactivation (reverse orientation) of neighboring genes in a Gal4-dependent manner. There are various collections of EP strains available allowing misexpression of a large number of fly genes [45,46]. So-called **RNAi lines** express short inverted repeat sequences under UAS control. The sequence of the inverted repeat corresponds to an endogenous gene. Gal4-dependent expression of the inverted repeat results in the formation short hairpin RNAs (shRNAs). The presence of shRNAs initiates a series of cellular mechanisms eventually resulting in silencing of the corresponding endogenous gene by RNA interference (RNAi) [47].

A more direct approach to investigate Aβ42-induced toxicity was used by Crowther and co-workers [34]. They fused Aβ40/42 peptides to the signal peptide of endogenous *Drosophila necrotic* gene sequence ensuring secretion [34]. Using the UAS/Gal4 inducible gene expression system (Figure 1), the authors generated transgenic flies allowing the spatiotemporal expression of Aβ40 and Aβ42. As the expressed Aβ40/42 correspond to the peptides generated by amyloidogenic processing of APP, influences that might result from APP processing are avoided. These flies have the major advantage of a direct assessment of Aβ toxicity.

Neuronal expression of Aβ42 caused neurotoxicity, locomotion defects and reduced lifespan. Moreover, intra- and extracellular accumulation of Aβ42 peptides was observed. Overexpression of Aβ42[E22G], known to increase the rate of Aβ42 aggregation [7], exacerbated the observed phenotypes [34]. Extensive investigation of molecular mechanisms leading to changes in synaptic transmission and protein composition at the presynaptic active zone revealed that Aβ42 expression affected axonal transport of mitochondria and resulted in depletion of mitochondria from the presynaptic active zone [48]. Intraneural accumulation of Aβ42 was shown to reduce synaptic vesicle release probability prior to bouton loss [49]. Patch clamp analysis revealed a depression of cholinergic synapses upon Aβ42 expression. Moreover, expression of a familial AD-linked mutant variant Aβ[E22G] caused an increased aggregation of the Aβ42 peptide [50].

Finelli and co-workers established fly lines expressing fully processed, secreted Aβ peptides [35]. The generated transgenes allowed in-depth analysis of Aβ accumulation as overexpression of human Aβ40 and Aβ42 peptides can be induced in a variety of cell types including neuronal cells. Both peptides accumulated in the fly brain but only Aβ42 formed deposits [51]. Consequently, only Aβ42 expressing flies show age-dependent and dose-dependent neurodegeneration. In these flies, short-term memory was impaired, obvious locomotor deficits appeared in aged flies and survival was reduced [37].

As memory loss is a well-known feature of AD in humans, memory assessment is widely used as an adequate tool to identify factors involved in Aβ42 pathomechanisms. Recently, excess epidermal growth factor receptor (EGFR) was shown to enhance short-term memory loss in flies concomitantly expressing Aβ42. The detrimental effect of EGFR overexpression on Aβ42-induced memory loss was verified by the application of known EGFR inhibitors, e.g. gefitinib and erlotinib. Both drugs are normally used in clinical cancer therapy, but were able to prevent Aβ42-induced memory loss in flies. Interestingly, also memantine, a drug that is already used to treat dementia in AD patients, prevented memory loss induced by Aβ42 expression in flies [52]. Positive effects of the mentioned drugs were also evident in double transgenic AD mice overexpressing two mutated AD-linked transgenes (APPswe/PSEN1dE9) [52,53]. Thus, results from invertebrate models systems might be well transferred to higher organisms.

Accumulating evidence suggests that impairment of metal homoeostasis is an important factor in AD pathogenesis. Levels of redox active metal ions such as copper, zinc and iron are elevated in amyloid plaques of AD patients [54]. Furthermore, it is known that presence of metals can promote Aβ aggregate formation *in vitro* and chelating agents are able to dissolve Aβ plaques in *post mortem* AD brains [55,56].

*Drosophila* models for AD proved to be a useful tool to investigate the influence of different metal ions on Aβ-induced neurodegeneration [57-61]. By feeding Aβ42 expressing flies with copper or zinc supplemented food the Aβ42-induced phenotypes such as REP decreased survival and locomotor defects were enhanced. In contrast, food supplemented with metal-chelating substances suppressed these phenotypes [57]. Genetic manipulation of metal homeostasis further underlined the role of zinc and copper levels in Aβ42-induced toxicity [57-59]. For example, overexpression of MTF-1, a highly conserved transcription factor inducing expression of several metal ion scavenger proteins, was shown to effectively protect from detrimental effects of Aß42 in flies [57]. Furthermore, genetic inhibition of two copper-importers (Ctr1C and Ctr1B) ameliorated Aß42-induced neurodegenerative phenotypes while lowering copper load in the fly brain [58]. A study focusing on zinc as another redox active metal and its modulation of Aβ42-induced phenotypes basically showed the same [59]. Genetic downregulation of the expression of the zinc importer dZip1 consistently suppressed Aβ42-induced brain vacuolization, locomotor defects and reduced lifespan, while overexpression had the opposite effect [59]. Furthermore, the authors were able to show an effect of zinc deposition on the accumulation of Aβ fibrils in *Drosophila* brains and a beneficial effect of dZip1 knockdown on Aβ-induced early memory loss [59].

While findings about the detrimental effects of metal ion-Aβ complexes find a growing consent, not much is known about the specific mechanisms of metal ions in AD. The study of Liu *et al.* took a closer look on the biophysical particularities of the interaction between iron and Aβ peptides [60]. First, a connection between the presence of iron and modulation of Aβ42-induced toxicity was observed. Manipulation of the expression of iron-binding proteins like ferritin and feeding of iron-specific chelating agents altered Aβ42-induced toxicity [60]. Surprisingly, knockdown of ferritin did not reduce Aβ accumulation but efficiently suppressed Aβ42-induced toxicity [60]. Instead, biophysical techniques revealed that the presence of iron during Aβ42 aggregation altered the structure of Aβ fibrils delaying the formation of mature aggregates [60]. Cytotoxicity assays using human neuroblastoma SH-SY5Y cells indicated that the presence of iron during aggregate formation was contributing to Aβ toxicity rather than addition of iron after aggregate formation [60]. Thus, the authors conclude that modulation of the kinetics of Aβ aggregate formation by iron is important for the toxicity of Aβ42 peptides [60].

Besides the potential function of metal ions to act as seeds for Aβ accumulation, they might also play a role in the production of reactive oxygen species (ROS) *via* Fenton-like reactions. An unbiased screen identified many modifiers of Aβ42-induced toxicity that were implicated in redox regulation [61]. Overexpression of two subunits of ferritin, a highly conserved protein with a strong antioxidant potential, efficiently prolonged the lifespan of Aβ42 expressing flies and simultaneously reduced the oxidative damage in fly brains [61]. Thus, sequestration of free radicals by ferroxidase activity might be a beneficial mechanism protecting from oxidative stress originating from the redox potential of Aβ peptides in the *Drosophila* model for Aβ42-induced toxicity [61].

Further adding to the topic of metal ions interacting with Aβ peptides is a study about intrinsic toxicity of aluminum [62]. Typical neurodegenerative phenotypes like reduced lifespan, locomotor deficits, olfactory learning abnormalities and vacuolization of the brain were observed after feeding *Drosophila* with excess aluminum [62]. Aluminum overload was shown to increase iron levels while simultaneously generating ROS. However, no direct link could be established between both processes [62]. Interestingly, expression of Aβ peptides or Tau did not modulate the Al-induced neurotoxicity [62]. This study indicates that heavy metal ions can exert neurotoxic effects *per se* and it remains to be elucidated if these mechanisms are the cause or consequence in the interplay between redox reactive metal ions, ROS generation and Aβ peptides.

Apart from Aβ42 deposits, AD in humans is characterized by intracellular neurofibrillary tangles composed of hyperphosphorylated Tau proteins. As the functional interactions between both AD lesions remain unclear, fly lines expressing Aβ42 were investigated for the formation of fibrillary structures with fly endogenous Tau protein. However, fibrillary structures composed of hyperphosphorylated Tau could not be detected in Aβ42-expressing flies using biochemical or histological methods [51].

### Drosophila models for Tau toxicity

Insoluble aggregates of the MT-associated protein Tau are a common feature of so-called tauopathies like frontotemporal dementia with parkinsonism linked to chromosome 17 (FTDP-17), progressive supranuclear palsy and Pick’s disease and others [63]. Central feature of tauopathies is the presence of paired helical filaments, which assemble into intracellular neurofibrillary tangles in affected tissues [64]. Several disease-linked mutations in the Tau gene affect correct splicing of its MT binding sites, thus enhancing abnormal phosphorylation and detachment of the protein. Both steps are believed to be crucial in the process of forming paired helical filaments and higher order neurofibrillary tangles [65,66].

Overexpression of wild-type or mutant human Tau in the *Drosophila* nervous system caused vacuolization in the brain accompanied by pathologic phosphorylation status of Tau, although large filamentous aggregates were absent [64]. Nevertheless, immunostaining with antibodies detecting abnormal confirmation of Tau revealed a close association between areas of degeneration and abnormal Tau in flies. Moreover, the abundance of vacuolar lesions in the fly brain was first observed in Tau expressing tissue. In addition, neurodegeneration progressed with fly age and eventually resulted in early mortality. Furthermore, severity of phenotypes was enhanced by increasing Tau dosage or introducing mutant Tau isoforms, such as the V337M and R406W mutations associated with FTDP-17 [64]. In addition, targeted expression of either wild-type or mutant Tau in the retina caused alterations in external eye structures, characterized by size reduction and rough appearance. The so-called rough eye phenotype (REP) correlates with the loss of retinal cells including photoreceptors [63,64,67,68]. Detailed analysis revealed that Tau overexpression caused degeneration of photoreceptor axons, evident by the appearance of vacuoles in the medulla, the projection target of photoreceptor axons [63]. Such REPs are frequently used to screen for genetic interactions (see Table 2). In such an approach the fly ortholog of glycogen synthase kinase 3β (GSK3β) was identified to interfere with Tau-induced toxicity. Interestingly, the Tau-induced REP was suppressed in a GSK3β-deficient background and enhanced by GSK3β overexpression [68]. Detailed analysis showed that overexpression of GSK3β strongly increased pathogenic phosphorylation of Tau [68,71].

**Table 2:** **Overview of performed large-scale screens for modifiers of toxicity induced by expression of AD-linked genes in *Drosophila melanogaster***

**Transgene causing a REP**	**Screened library**	**Results**	**Reference**
hTau[V337M]	2,276 EP strains	• Kinases, phosphatases (CDK5, GSK3β, PAR1)	Shulman & Feany [69]
		• Apoptosis	
		• Novel: Ataxin 2, Fmr1	
hTau[V337M]	1,250 P-element strains	• Cytoskeletal components	Blard *et al.*[70]
		• Molecular chaperones	
		• Chromatin remodelling	
hTau[WT]	920 P-lethal strains	• Kinases, Phosphatases	Ambegaokar *et al.*[71]
	895 EY strains	• Autophagy/lysosomal	
		• RNA processing	
		• Chromatin regulation	
		• Cytoskeletal	
GMR > Aβ42	1,963 EP strains	• Secretory pathway	Cao *et al.*[72]
		• Cholesterol homeostasis	
		• Chromatin regulation	
Pan neural Arctic Aβ42 life span reduction	3,000 de novo insertions of transposable elements	• Fenton chemistry and oxidative stress are involved in AD pathology	Rival *et al*. [61]
		• Ferritin expression protects from β-amyloid toxicity	

The table lists only screens in which the fly was used as primary screening tool. Not listed are screens using other sources to gain candidates, later confirmed in flies.

In order to investigate the role of Tau phosphorylation and toxicity in more detail, several Tau variants with altered phosphorylation sites were generated [67,73,74]. Chatterjee *et al.* created fly lines expressing phosphorylation-resistant Tau variants by exchanging two (Tau^S2A^) or eleven (Tau^S11A^) putative serine-threonine phosphorylation sites with neutral alanine. These mutations prevented phosphorylation by protease activated receptor 1 (PAR-1) and GSK3β, respectively [67]. This allowed a thorough investigation of several Tau kinases in disease-related processes such as site-specific phosphorylation and changes in MT binding properties of Tau [67]. Interestingly, REP enhancement induced by overexpression of GSK3β was less pronounced in the Tau^S2A^ expressing fly compared to the wild-type Tau expressing fly although immunoblotting using phosphorylation site-specific Tau antibodies showed a higher degree of Tau phosphorylation. In contrast, Tau^S11A^ was resistant to GSK3β phosphorylation although GSK3β overexpression enhanced the Tau^S2A^-induced REP severity. Furthermore, neither Tau aggregation nor MT binding properties consistently correlated with REP [67]. These results uncouple Tau toxicity from sole phosphorylation and indicate Tau toxicity is partially independent of its phosphorylation state.

In addition, Iijima-Ando *et al.* generated another phosphorylation-resistant Tau variant Tau^S262A^[73]. Retinal coexpression of wild-type human Tau and DNA damage-activated checkpoint kinase 2 (Chk2) resulted in enhancement of the REP. In contrast, coexpression of Chk2 and Tau^S262A^ had no effect on eye surface integrity [73].

To determine the contribution of specific phosphorylation sites to Tau toxicity, Steinhilb *et al.* designed novel Tau transgenes [74]. By replacing serines of several disease-associated phosphorylation sites with alanine they created a phosphorylation-resistant variant (Tau^AP^) and by replacing serines with glutamines they mimicked a hyperphosphorylated state of Tau (Tau^E14^). The consequences are amelioration of Tau toxicity in flies expressing phospho-deficient Tau variant Tau^AP^ and exacerbation of Tau toxicity in flies expressing the phospho-mimetic Tau variant Tau^E14^[74]. However, mutation of individual serines of the respective phosphorylation sites did not result in a clear modulation of Tau toxicity indicating that multiple sites work in concert to confer to Tau toxicity [75].

Folwell and co-workers analyzed concomitant expression of Aβ42 and Tau in flies. In these flies, Aβ42 expression exacerbated Tau-induced neuronal dysfunction, axonal transport deficits and decreased survival [76]. The combinatorial expression of both pathological proteins Aβ42 and Tau in *Drosophila* seems to be a promising approach to investigate the synergistic effects at the level of genetic interactions.

### Large-scale screens in Drosophila

Low demand on care and easiness of handling predestine the fly to high-throughput screens *in vivo*. Adding to these advantages is the extraordinary large pool of available genetic instruments paired with simplicity of the genomic structure facilitating subsequent in-depth analysis.

**Figure 2:**
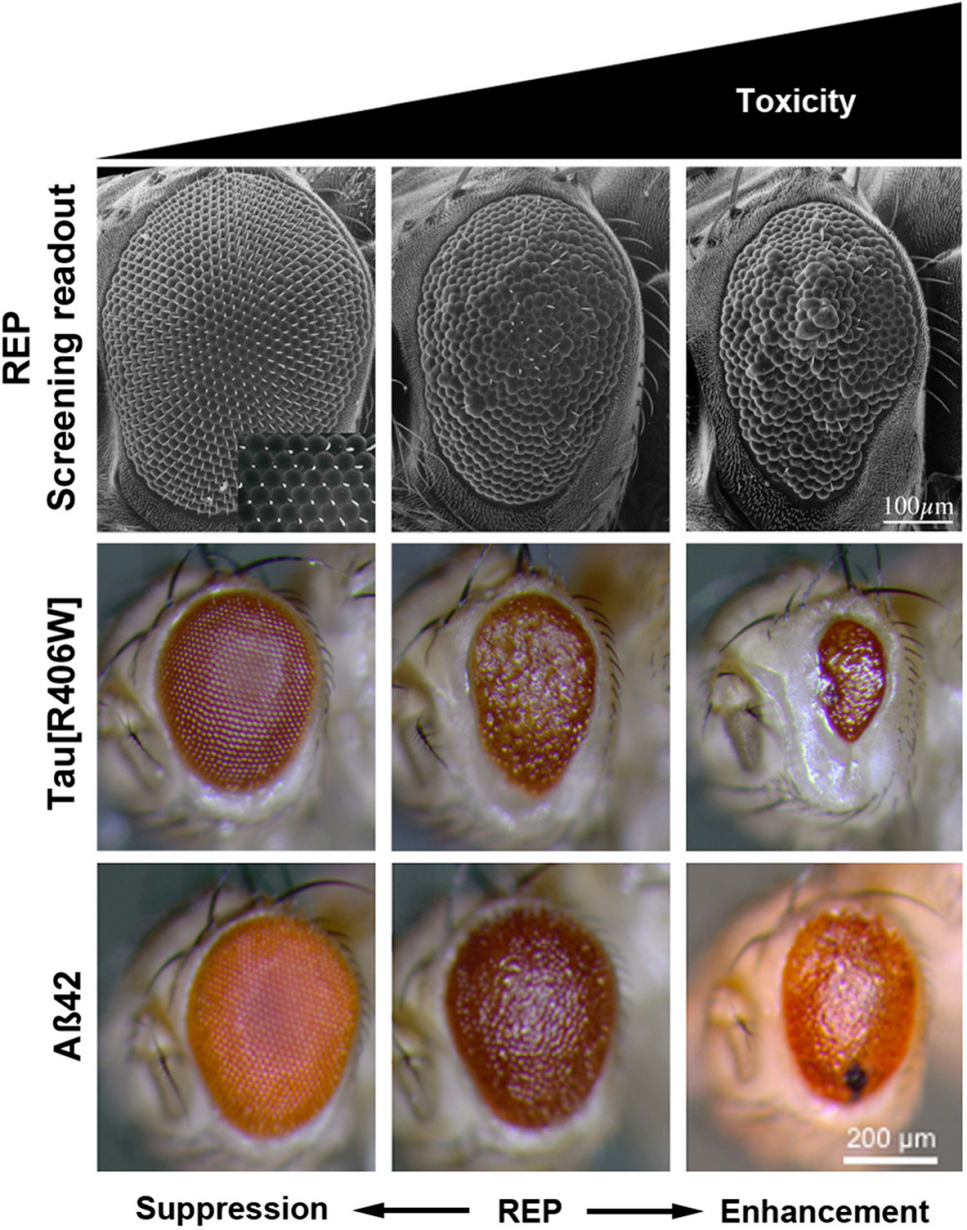
**Exemplified rough eye phenotypes (REP) used as readout for modifier screens.** Scanning electron micrographs (top) of fly eyes are shown. The *Drosophila* compound eye consists of a stereotypic array of about 800 omatidia (left). These hexagonal structures are highly ordered and display regular spacing of hairs called interomatidial bristles (inset). Expression of disease-linked proteins/peptides in the eye can cause a REP (middle). The rough appearance of the eye can be caused by loss of interomatidial bristles, fusion of omatidia, necrotic tissue, dints in the retina and is often accompanied by loss of pigmentation and reduced eye size. An enhancement in severity (left) is easily observable by more pronounced REP characteristics. Usually, such REPs are sensitive towards genetic interactions, causing either a suppression (left) or an enhancement (right), changing the overall eye appearance towards a more wild-type like appearance (suppression) or by increasing the rough appearance of the eye (enhancement), respectively. Exemplary light micrographs show REPs induced by expression of either Tau[R406W] (middle) or Aβ42 (bottom). These REPs are sensitive towards genetic modification like suppression (left) and enhancement (right) and can be/have been used for screening approaches.

Up to now unbiased screens in *Drosophila* were performed utilizing the above-described tools and provided valuable insights into AD pathomechanisms (see Table 2) [69-72]. REPs induced by expression of toxic gene products in the *Drosophila* compound eye represent an easy to score read-out for genetic modifier screens. The fly eye is a neuronal structure and REPs are highly sensitive to genetic modification. Changes in REP severity usually coincide with changes in photoreceptor degeneration, thus changes in neuronal decline can be investigated by light microscopy (Figure 2).

Shulman and Feany conducted the first large-scale screen in *Drosophila* for genetic modifiers of toxicity induced by expression of human Tau [69]. In their screen, the authors used the fact that eye-specific expression of a FTLD-linked Tau variant (Tau[V337M]) induced a moderate REP. To facilitate identification of enhancers and suppressors, flies with the Tau-dependent REP were crossbred with a collection of 2,276 enhancer promoter (EP) insertion-carrying flies. These files contain random insertions of EP-elements, which can be used to misexpress endogenous fly genes (Figure 1) [45]. EP-elements contain UAS sites allowing the Gal4-induced transcription of open reading frames in the vicinity of insertion. Depending on the orientation of the EP-element in relation to the open reading frame, Gal4 induces either ectopic overexpression or inactivation of the gene by RNA interference (RNAi) [45]. After comprehensive validation of identified candidates they were functionally classified. The largest group of modifiers were kinases and phosphatases. Among these kinases were *Drosophila* orthologs of known Tau kinases such as cyclin-dependent kinase 5 (CDK5) and GSK3β. Accordingly, these results confirmed the reliability of the screening approach and emphasizes the critical role of Tau phosphorylation for toxicity [69].

Using the same transgenic fly line expressing human Tau [V337M], Blard *et al.* screened a different collection of 1,250 EP-element containing fly lines [70]. According to the differences in fly lines and the low percentage of whole genome coverage, there was little overlap between identified modifiers from this screen compared to the screen by Shulman and Feany. Blard *et al.* identified several components of the cytoskeleton as modifiers of Tau-induced REP. In addition, the Tau-induced disruption of the MT network at nerve terminals was identified as key event leading to Tau-induced neurodegeneration [70].

The most recent large-scale screen for modifiers of Tau toxicity was performed by Ambegaokar *et al.*[71]. In their screen, the authors used a fly line expressing wild-type human Tau in the fly eye. This fly line also exhibited an intermediate REP, which was suitable to identify both enhancer and suppressors. The authors screened two independent collections of fly lines. The first contains roughly 1,000 lethal loss-of-function alleles caused by P-element insertion in essential genes. The second collection contained 900 lines with random insertions of EY-elements. These EY-elements are very similar to EP-elements and also contain UAS sites. Once Gal4 is present, this can result in overexpression or RNAi-mediated silencing of genes in close vicinity to the insertion site of the element (Figure 1). In their screen, Ambegaokar and co-workers identified known interactors of Tau toxicity such as the *Drosophila* ortholog of GSK3β. This can be regarded as validation of the screen and suggests that identified modifiers could be relevant to disease. Comprehensive analysis of identified modifiers using computational network approach revealed a broad range of functional classes including kinases, cytoskeletal components as expected but also mechanisms not yet associated to Tau toxicity such as RNA metabolism or chromatin interaction [71]. Furthermore, the authors found that differences in Tau phosphorylation did not correlate with changes in Tau toxicity [71].

Only few large-scale screens have been published identifying genetic modifiers of Aβ42-induced toxicity (see Table 2 and [61,72]). Cao *et al*. screened a collection of EP-element carrying fly lines for modification of Aβ42-induced REP in *Drosophila*[72]. Modifiers identified in this screen comprise loss-of-function alleles widely involved in cell compartment trafficking pathways leading to the conclusion that proper function of endocytosis and vesicular trafficking is critical to protect the cell from Aβ42-induced toxicity. In addition, a reasonable number of candidate genes involved in secretory pathways were identified. Thus, the authors argue that proteolytic degradation of Aβ peptides during translocation by the secretory pathways might be a crucial pathomechanism in AD [72]. On the other hand, Rival and co-workers convincingly showed that Fenton chemistry and oxidative stress contribute to the toxicity of β-amyloid peptides in flies [61].

The combination of the Aβ42-induced REP with the utilization of RNAi allows for an unbiased screen targeting known open reading frames of the *Drosophila* genome. Using an inducible short hairpin RNA (shRNA) expressing fly line, the RNAi effect can be activated in a spatio-temporal manner (Figure 1). Recently, an *in vivo* RNAi library was generated utilizing the UAS/Gal4 system to control shRNA expression [47].

The RNAi library has been extensively used for genome-wide, large-scale screens to identify genetic modifiers of basic cellular mechanisms [77-79]. However, published data regarding the above-described Aβ42 toxicity models are surprisingly scarce [72,80]. Nevertheless, this approach has been used to find genetic modifiers of Ataxin-3-derived polyglutamine-induced toxicity [81]. The analysis yielded a large number of genetic modifiers that imply involvement of multiple processes in polyglutamine toxicity.

To aid the understanding of mechanisms leading to AD, we performed a genome-wide screen for modifiers of Aβ42-induced neurodegeneration [82]. By combining eye-specific RNAi-mediated knockdown of single *Drosophila* genes and concomitant Aβ42 expression, genetic interactors modulating Aβ42-induced REP were identified and were assigned to cellular pathways contributing to Aβ42 toxicity. To prove adaptability of the performed screen, we tested RNAi lines targeting corresponding *Drosophila* orthologs of known susceptibility genes identified by genome-wide association studies (GWAS) for their ability to modulate the Aβ42-induced REP. Preliminary results indicate low conformity between the effects of RNAi-mediated knockdown of susceptibility genes and enhancement or suppression of Aβ42-induced REP (unpublished results). One way to explain this might be the redundancy of affected pathways. Another possibility might be low penetrance of the RNAi effect, although the majority of the RNAi library was tested for effective silencing of targeted genes [47]. Still, AD is not a monogenic disease and application of GWAS to identify human risk factors failed to find new major genes relevant to all AD patients [83]. In addition, we conducted a very similar screen to identify modifiers of Tau[R406W]-induced neurodegeneration. To our surprise, in this screen we only identified a very small amount of modifiers (less than 100 out of roughly 8,000 screened RNAi lines modified the Tau[R406W]-induced REP). Among the few candidates were members of the dynein/dynactin complex. As silencing members of the dynein/dynactin complex enhanced the Tau[R406W]-induced toxicity, an impaired retrograde axonal transport seems to contribute to Tau[R406W]-induced toxicity (to be published elsewhere).

### Perspectives and conclusion

*Drosophila melanogaster* is a useful *in vivo* tool to analyze pathomechanisms in AD. For example, aggregation of Aβ42 can be easily determined in flies. Thus, large collections of small compounds can be screened for their potency to inhibit Aβ peptide aggregation [80]. Recently, a compound (D737) was identified that effectively inhibited fibril formation *in vitro.* Administration of this compound to flies prevented early death usually observed after Aβ42 expression [80]. Such *in vivo* approaches might help in drug development not only in case of AD, but also in the context of other (neurodegenerative) diseases.

Furthermore, transgenic fly lines can be used to prove efficiency of β-secretase steady-state inhibitors [84]. β-Secretase activity is the rate-limiting step during amyloidogenic processing leading to the generation of pathogenic Aβ peptides. Thus, β-secretase activity is a preferred target for the development of pharmacological therapies against AD. *In vitro* assays proved the activity of several engineered β-secretase inhibitors but many failed in cellular assays [85,86]. However, *in vivo* the endosomal localization of β-secretase is essential for activity. Coupling of a sterol moiety to the inhibitor resulted in successful delivery to the endosomal membrane and efficient inhibition of β-secretase cleavage of APP in several cell lines [84]. Furthermore, inhibition of β-secretase activity by the sterol-coupled inhibitor was shown to be efficient *in vivo* using the triple transgenic fly line expressing hAPP, hBACE and dPsn created by Greeve *et al.*[36]. Transgenic larvae fed with the membrane-tethered steady-state inhibitor showed increased hatching rates compared to transgenic larvae fed with soluble inhibitor [84]. Thus, flies expressing disease-related transgenes might be very useful to prove hypotheses *in vivo* in a fast, effective and economic manner.

Despite the efforts of countless scientists worldwide to clarify the mechanisms underlying the most prevalent form of dementia, it is still not possible to cure AD. Until now therapies for AD have included only symptomatic treatment and there is not even any effective medication to stop disease progression. The mere number of hypotheses intending to explain the pathogenesis of AD hints at the general challenge this disease poses to modern science. The challenge now is to elucidate the contribution of AD-associated pathways with known effects to Aβ42-induced neurodegeneration and to differentiate the pathways modifying general neurodegenerative mechanisms from the ones that are unique to AD and thus provide a target for drug development.

## Ethical approval

Experimental research reported here was performed using insects (*Drosophilae*). Such research is exempt from regulations pertaining to ethical approvals and/or animal protection laws.

## Abbreviations

Aβ: Amyloid-β; AD: Alzheimer’s disease; APP: Amyloid precursor protein; BACE: β-site APP-cleaving enzyme; dAPPl: APP-like, *Drosophila melanogaster* ortholog of APP; dPsn: *Drosophila melanogaster* ortholog of presenilin; EGFR: Epidermal growth factor receptor; EP: Enhancer-promoter; FTDP-17: Frontotemporal dementia with parkinsonism linked to chromosome 17; GSK3β: Glycogen synthase kinase 3β; GWAS: Genome-wide association studies; MT: Microtubuli; PAR1: Protease activated receptor 1; REP: Rough eye phenotype; RNAi: RNA interference; shRNA: short hairpin RNA; UAS: Upstream activating sequence.

## Competing interests

The authors declare that they have no competing interests.

## Authors’ contributions

KP and AV wrote the manuscript. JBS wrote and drafted the manuscript. All authors read and approved the final manuscript.
